# Hepatoprotective Effect of Cranberry Nutraceutical Extract in Non-alcoholic Fatty Liver Model in Rats: Impact on Insulin Resistance and Nrf-2 Expression

**DOI:** 10.3389/fphar.2020.00218

**Published:** 2020-03-18

**Authors:** Safaa A. Faheem, Noha M. Saeed, Reem N. El-Naga, Iriny M. Ayoub, Samar S. Azab

**Affiliations:** ^1^Department of Pharmacology and Toxicology, Faculty of Pharmacy, Egyptian Russian University, Cairo, Egypt; ^2^Department of Pharmacology and Toxicology, Faculty of Pharmacy, Ain Shams University, Cairo, Egypt; ^3^Department of Pharmacognosy, Faculty of Pharmacy, Ain Shams University, Cairo, Egypt

**Keywords:** cranberry nutraceutical, NAFLD, liver fibrosis, Nrf-2, insulin sensitivity

## Abstract

**Background:**

Non-alcoholic fatty liver disease (NAFLD) is a pathological accumulation of triglycerides (TGs) in the hepatocyte in the absence of alcohol intake. Untreated NAFLD is expected to progress into liver fibrosis. Cranberry is rich in polyphenols with antioxidant and anti-inflammatory activities.

**Hypothesis:**

The present study was performed to evaluate our hypothesis of the possible anti-fibrotic effect of cranberry nutraceuticals in a high fat cholesterol diet induced (HFCD)-NAFLD in rats, focusing on improving insulin sensitivity and modulating the expression of nuclear factor erythroid-2-related factor-2 (Nrf2) (a transcription factor responsible for regulating cellular redox balance).

**Method:**

Male albino wistar rats (12 weeks) received HFCD and/or cranberry (50 and 100 mg/kg/day, three times/week) orally for 8 consecutive weeks.

**Results:**

In comparison to the HFCD group, cranberry treated groups (50 and 100 mg/kg) showed marked hepatoprotection, where it significantly decreased liver enzymes (alanine transaminases by 49 and 64% and aspartate transaminases by 45 and 64%; respectively), TGs, and ameliorated the histopathological alterations (such as inflammatory cells infiltration and ballooning degeneration) induced by HFCD. Cranberry also alleviated oxidative stress (malondialdehyde, glutathione, catalase and superoxide dismutase) and inflammation (tumor necrosis factor- alpha, interleukine-6 and nuclear factor kappa-b) and significantly reduced the HOMA-IR and TyG index. On the other hand, cranberry treated groups (50 and 100 mg/kg) showed a marked increase in the expression of adiponectin, by 8 and 13-fold, insulin receptor substrate-2 by 21 and 79%, and Nrf2 by 13 and 61%, respectively. Notably, cranberry significantly reduced the fibrotic markers, TGF–β and α-SMA expression and collagen deposition.

**Conclusion:**

The present study showed that cranberry significantly attenuated NAFLD, in a dose dependent manner, which could be partially recognized by its antioxidant, anti-inflammatory activities, and its ability to improve insulin sensitivity. Notably, our study proves for the first time that the anti-fibrotic activity of cranberry is promising.

## Introduction

Metabolic syndrome (MetS) is considered to be one of the main sources of morbidity and mortality in different countries (Simons et al., 2011). It is described as several disorders including obesity, dyslipidemia, hypertension, and insulin resistance (IR) (Reaven, 2002). The growing epidemic of MetS has been associated with a hepatic manifestation represented as non-alcoholic fatty liver disease (NAFLD) (Angulo, 2002; Marchesini et al., 2003), which is considered to be a variety of liver disorders progressing from triglyceride’s (TGs) accumulation in the hepatocyte (steatosis) to non-alcoholic steatohepatitis (NASH), developing to fibrosis, cirrhosis, and finally to hepatocellular carcinoma (Moore, 2010; Tarantino et al., 2012). In fact, recent research states that one of the most important causes of hepatocellular carcinoma is the increased prevalence of NAFLD (Dhamija et al., 2019).

The hypothesis of NAFLD progression is based on multiple hits, the first hypothesis is the increase of TGs in the hepatocyte causing steatosis which results from increased peripheral lipolysis and leads to a high level of circulating free fatty acid (FFA) (Eguchi et al., 2006). Although fat accumulation in liver is a benign process, when the detoxification capacity of the liver fails, lipotoxicity of the liver begins and hepatic inflammation occurs. Moreover, Hyperinsulinemia and hyperglycemia cause an increase in *de novo* lipogenesis (DNL) (the conversion of carbohydrates to fatty acids) (Postic and Girard, 2008), which is responsible for decreasing the levels of the anti-inflammatory cytokine, adiponectin (ADP) (Bajaj et al., 2004).

ADP represents an anti-diabetic, anti-inflammatory adipocytokine which induces an extracellular Ca^+2^ influx that in turn, activates sirtuin. It has been widely stated that NAFLD is usually associated with a low level of adiponectin and a low level of sirtuin1. Hepatocyte specific deletion of sirtuin1 not only leads to hepatic steatosis, but also hepatic inflammation. Moreover, adiponectin plays a protective role by reducing hepatic fat content and improving insulin sensitivity. Therefore, it might play an important role in preventing hepatic inflammation (Wolf et al., 2004).

Leptin: is an adipocytokine released in response to expansion of adipocyte by TGs. It acts by reducing fat content in peripheral organs and stimulating fatty acid oxidation. NAFLD is associated with a low leptin level which in turn, induces hepatic inflammation. This cytokine inhibits the hepatic expression of tumor necrosis factor-alpha (TNF-α), thereby decreasing its plasma concentration (Anstee and Goldin, 2006).

Additionally, DNL down-regulates the protective nuclear factor erythroid-2-related factor-2 (Nrf2), which shows a vital protective role as an anti-inflammatory and anti-fibrotic factor. In addition, Nrf-2 has a cellular defense mechanism which works by increasing the expression of the anti-oxidant genes and also protecting the hepatocyte from damage caused by the accretion of fat (Kay et al., 2011).

Furthermore, the increase in the mitochondrial β-oxidation of FFA, as a result of the increased accretion of fat in the hepatocyte, induces the reactive oxygen species (ROS) leading to NASH and fibrosis (Day and James, 1998; Day, 2002). The overall surge of the oxidative stress leads to lipid peroxidation which, subsequently, increases nuclear factor-kappa B (NF-κB) expression. NF-κB is known to be a transcription factor that impels the secretion of pro-inflammatory cytokines such as TNF-α and interleukin-6 (IL-6). The recruited inflammation in hepatic tissues induces transforming growth factor-beta (TGF-β) expression, which activates hepatic stellate cells (HSCs) leading to an increase in the expression of alpha-smooth muscle actin (α-SMA), the HSCs activated marker. All these events lead to collagen production and initiation of liver fibrosis (Browning and Horton, 2004). Besides all the previously mentioned signaling changes, the gut microbiota is a natural barrier that protects against the entrance and the spread of endotoxin and bacteria such as lipopolysaccharides (LPS) in the circulation. However, in NAFLD, this intestinal barrier fails, leading to the invasion of endotoxin and bacteria in the gastrointestinal tract, which subsequently reach hepatocyte tissues. Normally, the liver’s endothelial system is responsible for clearance of endotoxemia in the portal circulation, mainly Kupffer cells (Eslamparast et al., 2013), however, in the case of liver disorders, it fails to clear these endotoxins because the endotoxemic burden is higher. This in turn exerts pro-inflammatory signals mediated through toll-like receptors TLRs. The main inflammatory cascades include the c-Jun-N- terminal kinase and the NF-κB pathway (Baldwin, 1996).

Currently, there is no specific therapy for MetS or NAFLD, and only physical activity, weight loss, and life style modifications are useful (Asrih and Jornayvaz, 2014). The use of therapeutic agents, such as metformin, statin and other anti-oxidants, have shown promising benefits in decreasing the incidence of the disease (Younossi, 2008). In 1989, nutraceutical was coined from pharmaceutical and nutrition, by Stephen Defelice. According to him, a nutraceutical is considered to be any element that is a food or a part of food and has health or medical benefits, including the prevention and treatment of disease. For instance, blueberry nutraceutical extract was shown to protect against cardiac hypertrophy in an experimental model (Eladwy et al., 2018). Nutraceutical interventions could be potential therapies for NAFLD *via* improving insulin sensitivity which subsequently improves hepatic steatosis, oxidative stress, hepatic inflammation, and finally hepatic fibrosis (Del Ben et al., 2017). Interestingly, cranberry (Vaccinium macrocarpon) is considered to be an important nutraceutical source of phytochemicals, mainly polyphenols (Blumberg et al., 2013), which has been proven to have an anti-oxidant effect (Neto, 2007). In addition, cranberry showed anti-carcinogenic (Déziel et al., 2012), anti-inflammatory (Kivimäki et al., 2012), and anti-microbial effects (Uberos et al., 2011). The intake of cranberry has been reported to improve oxidative stress, hyperglycemia, as well as dyslipidemia in individuals with MetS (Shidfar et al., 2012). Furthermore, cranberry administration significantly improved insulin sensitivity (Anhê et al., 2014). However, the role of cranberry in mitigating liver fibrosis was not studied before. Accordingly, this study was conducted to investigate for the first time the possible anti-fibrotic effect of cranberry nutraceutical in a model of high fat diet induced NAFLD and liver fibrosis in rats. Moreover, our aim was clarified *via* studying different oxidative, inflammatory markers, as well as insulin sensitivity and signaling.

## Materials and Methods

### Drugs and Chemicals

Cranberry nutraceutical was obtained from Bulk supplements, Henderson, Unitd States (186c1025), and its composition was: organic acids 30%, sugars 39.4%, total polyphenols 10.6%, anthocyanins 1.2%, and proanthocyanidins 5.5%. Cranberry nutraceutical was diluted in water (40 mg/ml) (Elberry et al., 2010). The doses of Cranberry were selected based on a pilot study as well as a previous study (Elberry et al., 2010). Cholesterol, casein, and methionine were obtained from Medico Pharma, Cairo, Egypt.

For the characterization of the plant, methanol and formic acid (LC/MS grade) were supplied by Thermo Fisher Scientific (Thermo Fisher Scientific, United Kingdom). LC/MS grade water (Millipore, Milford, MA, United States) was used for LC/MS analysis. HPLC analytical grade methanol and Folin Ciocalteu reagent were obtained from Sigma-Aldrich (St. Louis, MO, United States). Aluminum chloride, sodium carbonate and potassium acetate were supplied by Al-Nasr Co., Egypt. Reference standards of gallic acid and quercetin were obtained from Sigma-Aldrich (St. Louis, MO, United States). All other chemicals were of highest available commercial grade.

### Ethics Statement

Animal care and all experimental protocols were approved and conducted in accordance with the guidelines approved by the Research Ethics Committee of Ain Shams University (REC-ASU), Egypt (Serial number of the protocol: Masters 116).

### UPLC-ESI-MS/MS Analysis of Cranberry Extract

Chromatographic analysis was achieved using an Acquity UPLC system (Waters, Milford, MA, United States) equipped with a reversed phase column (ACQUITY UPLC- BEH, C18, 50 × 2.1 mm, 1.7 μm particle size). Gradient elution was applied using a binary mixture of mobile phase A (H_2_O: HCOOH, 99.9:0.1, v/v) and mobile phase B (CH_3_OH: HCOOH, 99.9:0.1, v/v). The following gradient was applied at a flow rate of 0.2 ml/min: 0–2 min, isocratic 10% B; 2–5 min, linear from 10 to 30% B; 5–15 min, gradient from 30 to 70% B; 15–22 min, gradient from 70 to 90% B; 22–25 min, isocratic 90% B; 25–26 min, gradient from 90 to 100% B; 26–29 min, isocratic 100% B, 29–32 min, gradient from 100 to 10% B. The sample was prepared at a concentration of 1 mg/ml in methanol (HPLC grade) and the injection volume was 10 μl.

Eluted compounds were detected using a XEVO TQD triple quadrupole mass spectrometer (Waters Corporation, Milford, MA01757, United States) equipped with an electrospray ion source. The following instrument settings were applied: nebulizer gas, N2; drying gas, N2; cone voltage, 30eV; capillary voltage, 3kV; source temperature, 150°C; desolvation temperature, 440°C; cone gas flow, 50L/h; and desolvation gas flow, 900L/h. Mass spectra were acquired in both negative and positive ion modes between *m/z* 100–1000. Chromatographic peaks and mass spectra were processed using Maslynx 4.1 software. Compounds were characterized by comparison of their mass spectra and MS/MS fragmentation patterns with reference literature.

### Total Flavonoids Quantification

A spectrophotometric method was adopted for the quantification of flavonoids in cranberry extract using aluminum chloride reagent (Geissman, 1962). Cranberry extract was dissolved in methanol to prepare 1 mg/ml solution. An aliquot (1 ml) of cranberry extract was mixed with 1 ml of 10% AlCl_3_, 1.5 ml methanol, 0.1 ml of 1 M potassium acetate solution and 2.8 ml distilled water. The reaction mixture was mixed well and allowed to stand for 30 min at room temperature. Absorbance was measured at 415 nm. Quercetin was used as a standard to construct the calibration curve at a concentration range of 5–100 μg/ml. The assay was performed in triplicate. The total flavonoid content expressed in μg quercetin equivalent (QE)/mg cranberry extract was assessed.

### Total Phenolics Quantification

A spectrophotometric method was conducted for the quantification of total phenolics in cranberry extract using Folin–Ciocalteau reagent. Cranberry extract at a concentration of 1 mg/ml was prepared in water. Folin Ciocalteau reagent (1 ml) was added to 1 ml of the extract solution and 10 ml of distilled water in a volumetric flask. Contents were mixed, and after 5 min, 4 ml of a 20% Na_2_CO_3_ solution were added, and the volume was adjusted to 25 ml by the addition of distilled water and then, incubated at room temperature for 30 min. Absorbance was measured at 765 nm. Gallic acid was used as a standard to construct the calibration curve at concentrations of 80–280 μg/ml. The assay was carried out in triplicate. Total phenolic content was calculated as μg of a gallic acid equivalent (GAE)/mg of cranberry extract.

### Animal

Male albino wistar rats (12 weeks) (100–150 g) were attained from the Holding Company for Biological Products & Vaccines VACCERA, Egypt. The normal chow was obtained from Meladco for Animal Food, Egypt. The HFCD contained in grams per 100 g, 58 rat chow diet, 1 cholesterol, 10 corn oil, 18 butter, 5 caseins, 0.2 methionine, 5 sucrose, 0.8 vitamin mixture, and 2 mineral mixtures, while the normal diet contained in gram per 100 g, 48.8 carbohydrates, 21 proteins, 3 fats, 0.8 calcium, 0.4 phosphorus, 5 fibers, 18 moistures, 8 Ash. Pellets and tap water were provided *ad libitum*. The temperature was kept at 25°C. A 12/12 h light/dark cycle was preserved. Rats were acclimatized for 2 weeks to the lab conditions.

### Experimental Design

There are several animal models for NAFLD/NASH but, we chose the HFCD model because this is the only model that causes obesity (Hebbard and George, 2011). Fifty Male albino Wistar rats were randomly divided into five groups (*n* = 10). Group (1) was fed a normal rat chow diet for 8 weeks and received 1 ml/kg distilled water orally three times weekly and was considered the control group. Group (2) was fed a high-fat cholesterol diet (HFCD) for 8 weeks and received 1 ml/kg distilled water orally three times weekly for 8 weeks and was considered the HFCD group. Group (3) was fed a HFCD for 8 weeks and received cranberry (50 mg/kg/day) orally three times weekly for 8 weeks and was considered the cranberry 50/HFCD treated group. Group (4) was fed a HFCD for 8 weeks and received cranberry (100 mg/kg/day) orally three times weekly for 8 weeks and was considered the cranberry 100/HFCD treated group. Group (5) was fed a normal rat chow diet and received cranberry (100 mg/kg/day) orally three times weekly for 8 weeks and was considered the cranberry-only treated group. The weight of each animal was recorded at the beginning of each week. At the end of the experiment, the fasting blood glucose was determined and the final body weight of all animals was recorded. Blood samples were also collected from the different experimental groups and centrifuged to separate serum, which was then divided into aliquots and stored at −80°C until biochemical analysis. Animals were sacrificed and the liver was dissected out and weighed to calculate liver index. One part of the liver was stored at −80°C for the biochemical analysis. The other parts were kept in 10% buffered formaldehyde for immunohistochemical and histopathological examination.

### Assessment of Hepatotoxicity Markers

Serum alanine transaminase (ALT) and aspartate transaminase (AST) were assessed calorimetrically according to the manufacturer’s instructions (TECO Diagnostic, Anaheim, CA, United States). Results were expressed as U/L. Serum TGs levels were assessed using available enzymatic assay kits according to the manufacturer’s instructions (Spectrum Diagnostics, Cairo, Egypt). Results were expressed as mg/dl.

### Assessment of Oxidative Stress Marker

Lipid peroxidation was measured by assessing the reactive substances thiobarbituric acid tissue levels, measured as malondialdehyde (MDA), according to the manufacturer’s instructions (San Diego, CA, United States). Results were expressed as μmol/mg protein. Tissue levels of superoxide dismutase (SOD), Glutathione (GSH) depletion and catalase activity were estimated according to the manufacturer’s instructions (CUSABIO, Wuhan, China), (blue gene, Shanghai, China) and (Eiaab, Wuhan, China), respectively.

### Assessment of Insulin Resistance by HOMA-IR and TyG Index

We uses a validated one-touch basic glucose measurement system (Bionime Rightest GM300 blood glucose meter; Amazon, Taiwan) to measure blood glucose levels. Insulin levels were estimated by enzyme linked immunosorbent assay (ELISA) according to the manufacturer’s instructions (San Diego, CA, United States). Homeostasis model assessment for insulin resistance (HOMA-IR) was calculated using the following formula: (Insulin (μU/ml) × glucose (nM)/22.5) and used as an index for insulin resistance. TyG index was calculated with the following formula: Ln [(fasting triglycerides) (mg/dl) × fasting glucose (mg/dl)/2].

### Enzyme- Linked Immunosorbent Assay (ELISA) Assessment of TNF-α, IL-6, NF-κB, and ADP

The serum levels of the inflammatory markers, TNF-α and IL-6, were estimated according to the manufacturer’s instructions (Ray Biotech, Norcross, Georgia, United States), (Cusabio, Wuhan, China), respectively. Results were expressed as pg/ml. NF-κB levels in liver homogenate were estimated according to the manufacturer’s instructions (Eiaab, Wuhan, China). Level of NF-κB was expressed as ng/mg protein. The Cytoprotective ADP level was estimated in liver homogenate according to the manufacturer’s instructions (CUSABIO, Wuhan, China).

### Western Blot Analysis of Insulin Receptor Substrate-2 and Nrf-2 Expression

Insulin receptor substrate-2 (IRS-2) and nuclear Nrf-2 expression in liver homogenates were detected by western blot analysis. Whole-cell protein extracts were obtained after homogenization of liver tissue in ice-cold lysis buffer containing 62 mM Tris (pH 6.8), 10% glycerol, 2% sodium dodecyl sulfate (SDS). The protein concentration was determined using a Bio-Rad DC protein assay kit (Bio-Rad Laboratories, CA, United States). Proteins were separated in 10% polyacrylamide gel and electro-blotted on PVDF membranes (Bio-Rad Laboratories, CA, United States). The membranes were incubated with the rabbit primary antibody specific to the assessed proteins, followed by anti-rabbit secondary antibody-linked to horseradish peroxidase (HRP). Protein bands were visualized using enhanced luminol–based chemiluminescent (ECL Reagent, Abcam, MA, United States). The signal was captured on x-ray film and quantified using image analysis software (Image J, 1.46a, NIH, United States).

### Immunohistochemistry Analysis of Fibrotic Markers; TGF-β and α-SMA

The expression of α-SMA and TGF-β in the hepatocyte was shown by immunohistochemical analysis. Rat liver paraffin-embedded sections of 4 μm thickness were de-paraffinized in xylene and rehydrated in graded ethanol solutions to distilled water. Sections were then incubated with 5% bovine serum albumin in Tris buffered saline (TBS) for 2 h to block non-specific immunoreactions. Sections were then incubated with the primary antibody for TGF-β (Sigma-Aldrich Chemical Co., St. Louis, MO, United States), in a dilution of 1:50–1:100, and for α-SMA (Sigma-Aldrich Chemical Co., St. Louis, MO, United States), at 4°C overnight for immunostaining. After washing the sections with TBS, they were incubated with the secondary antibody for 1 h at room temperature. Sections were then washed and incubated with diaminobenzidine at room temperature. The slides were counterstained with hematoxylin. Positive immunoreactions were visualized under a light microscopy and quantified using image analysis software (Image J, 1.46a, NIH, United States).

### Biochemical Assessment of Collagen

The tissue levels of collagen were estimated according to the method of Woessner (1961). Results were expressed as mg/g tissue.

### Histopathological Examination

After sacrifice, liver tissues from the different experimental groups were fixed in 10% buffered formaldehyde. After fixation, 4 μm paraffin sections were stained with hematoxylin and eosin (H&E) as well as Masson’s trichrome. The slides were evaluated under the light microscope.

### Statistical Analysis

All data are expressed as mean ± S.E.M. We use one-way analysis of variance (ANOVA) followed by the Tukey-Kramer test for Statistical analysis differences among groups. The 0.05 level of probability was used as the criterion for significance. All statistical analysis was achieved using instant version 3 software package. Graphs were sketched using GraphPad Prism (ISI Software, United States) version 5 software.

## Results

### Metabolites Characterization by UPLC-ESI-MS/MS

**FIGURE 1:**
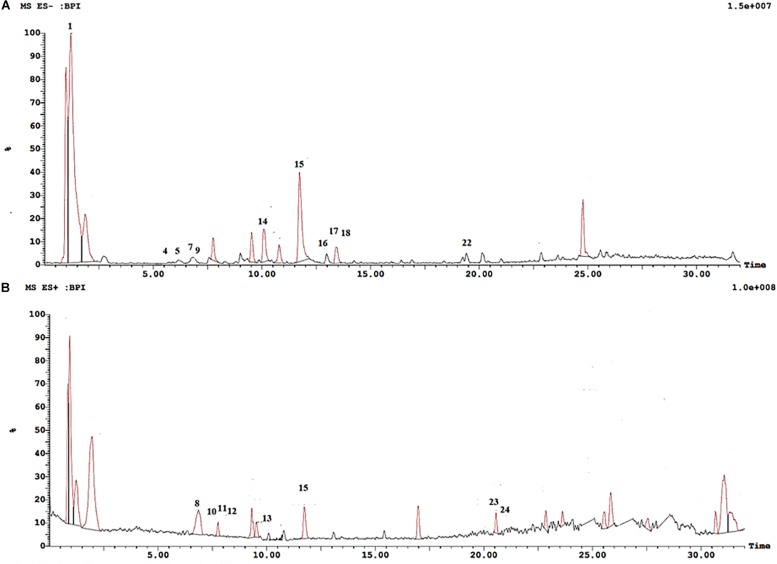
UPLC-ESI-MS chromatogram of cranberry extract. **(A)** UPLC-ESI-MS chromatogram of cranberry extract in negative ion mode. **(B)** UPLC-ESI-MS chromatogram of cranberry extract in positive ion mode.

Metabolite profiling of cranberry extract was performed using UPLC-ESI-MS/MS in both negative and positive ion modes (Figure 1). A total of 24 compounds were identified in the extract. Retention times, compound identities, molecular ions, and their observed fragment ions are displayed in Table 1. Peak assignments were based on comparing the mass fragmentation pattern of the eluted compounds with published data and online public databases.

**TABLE 1:** Compounds assigned in cranberry using UPLC-ESI-MS/MS in both negative and positive ion modes.

**Peak #**	**R*_*t*_* (min**	**Metabolite**	**[M-H]^–^**	**[M + H]^+^**	**MS^n^ ions (*m/z*)**	**References**
1	1.28	Citric acid	191		111	Fernández-Fernández et al., 2010
2	4.11	B-type proanthocyanidin dimer		561	427	Lin et al., 2014
3	5.40	Chlorogenic acid	353		191	Lin et al., 2014
4	6.17	p-Coumaric acid	163		119	Borges et al., 2009
5	6.38	B-type proanthocyanidin dimer		561	291, 276	Borges et al., 2009
6	6.64	Syringic acid	197		182	Lin et al., 2014
7	6.78	Cyanidin-3-*O*-hexoside		M^+^ 449	287	Borges et al., 2009
8	6.90	Citric acid (Isomer)	191		111	Flamini, 2003
9	7.46	Peonidin-3-*O*-hexoside		M^+^ 463	301	Fernández-Fernández et al., 2010
10	7.70	Peonidin-3-*O*-pentoside		M^+^ 433	301	Flamini, 2003
11	7.70	Malvidin-3-*O*-hexoside		M^+^ 493	331	Scharf et al., 2019
12	9.52	A-type procyanidin dimer		577	409, 287	Flamini, 2003
13	9.52	B-type proanthocyanidin dimer		593	277, 223, 219, 152	Lin et al., 2014
14	10.25	Myricetin	317		151	Scharf et al., 2019
15	11.73	Quercetin	301	303	179, 151	Scharf et al., 2019
16	13.25	Procyanidin trimer A type	863		804, 702, 603, 571	Lin et al., 2014
17	13.66	Syringetin	345		330, 315	Scharf et al., 2019
18	13.88	Isorhamnetin	315		300	Scharf et al., 2019
19	14.25	B-type procyanidin tetramer		1155	926, 741, 388, 287	Lin et al., 2014
20	15.48	B-type procyanidin tetramer	1153		890, 881, 569	Lin et al., 2014
21	16.28	B-type procyanidin pentamer		1443	579	Lin et al., 2014
22	20.19	B-type procyanidin tetramer	1153		863, 747, 231	Lin et al., 2014
23	21.03	B-type procyanidin hexamer		1731	577	Lin et al., 2014
24	22.58	B-type procyanidin hexamer		1731	577	Lin et al., 2014

Four major classes of natural products were detected in cranberry extract including phenolic acids, anthocyanins, flavonols, and proanthocyanidins. Chlorogenic acid (3), p-coumaric acid (4), and syringic acid (6) were the main phenolic acids detected in cranberry extract. Moreover, four anthocyanin glycosides were characterized. They were tentatively identified as cyanidin-3-*O*-hexoside (7), peonidin-3-*O*-hexoside (9), peonidin-3-*O*-pentoside (10), and malvidin-3-*O*-hexoside (11). Mass spectra of the identified compounds were consistent with published data in the literature (Flamini, 2003; Scharf et al., 2019).

Flavonoids are an important class of compounds present in cranberries. The identification of flavonoids herein was based on assigning the molecular ions and inspecting the tandem MS fragments. Myricetin (**14**), quercetin (**15**), syringetin (**17**), and isorhamnetin (**18**) were the major flavonols identified in the extract. Proanthocyanidins (PAs) are oligomers or polymers of flavan-3-ols, mainly (epi)catechins, attached through C4–C6 or C4–C8 single bond in B-type PAs, whereas, an additional ether bond (C2–O–C7 or C2–O–C5) bond occurs in type A PAs (Howell, 2007). Cranberry extract showed richness in proanthocyanidins, consisting predominantly of (epi)catechin units with varying degrees of polymerization. Some showed A-type linkage exhibiting [M + H]^+^ molecular ion at *m/z* 577 identified as A-type procyanidin dimer; and [M-H]^–^ a pseudo molecular ion at *m/z* 863 corresponding to A-type procyanidin trimer. Procyanidins are PAs formed only from epicatechin units, whereas those formed from two dissimilar units are termed proanthocyanidins (Lin et al., 2014). B-type proanthocyanidin dimer showed a quasimolecular ion at *m/z* 561 [M + H]^+^ and a fragment ion peak at *m/z* 427 [retroDiels-Alder rearrangement (RDA)]. B-type procyanidin tetramers, pentamers and hexamers were also identified exhibiting molecular ion peaks at *m/z* 1155, 1443, 1731, respectively (Table 1).

### Total Flavonoids and Total Phenolics Content

In this study, the total flavonoids and the total phenolic content in cranberry extract were quantified as quercetin and gallic acid equivalents, respectively. The total flavonoid content in cranberry extract was 2.54 ± 0.02 μg quercetin equivalent (QE)/mg cranberry extract. The total phenolic content in cranberry extract was found to be the 9.42 ± 0.5 μg gallic acid equivalent (GAE)/mg of cranberry extract. These results were consistent with previous studies (Mikulic-Petkovsek et al., 2012; Grace et al., 2013).

### The Effect of Cranberry Administration on Body Weight, Liver Index, Hepatic Enzymes, and TGs

Body weight was monitored throughout the experiment. After 8 weeks there was a significant rise in the body weight of rats fed HFCD – 9.1%, as compared to control values. In contrast, there was a significant decrease in the body weight of rats co-treated by CE50 and CE100 of 4 and 6%, respectively, as compared to the HFCD group. In addition, there was a significant rise in the liver index in HFCD group by 49%, as compared to the control group. On the contrary, the administration of cranberry, at doses of 50 and 100 mg/kg, significantly decreased this ratio by 16 and 22%, respectively, as compared to the HFCD group. Additionally, in the group that received a high fat diet, ALT and AST levels significantly rose by 4-fold, as compared to the control group. On the contrary, the administration of cranberry extract significantly decreased ALT and AST levels, in a dose dependent manner, as compared to the HFCD group. Additionally, TGs were significantly increased in the HFCD group by 2.1-fold, as compared to the control group. The administration of cranberry, at doses of 50 and100 mg/kg, significantly inhibited TGs levels by 38 and 58%, respectively, as compared to the HFCD group (Table 2).

**TABLE 2:** The effect of cranberry nutraceutical (50 and 100 mg/kg) on HOMA-IR, serum TG, liver index, ALT, AST, and tissue levels of ADP in HFCD-induced liver fibrosis in rats.

**Groups**	**HOMA-IR**	**TyG index**	**Serum TG (mg/dl**)	**Liver index (%)**	**ALT (IU/L)**	**AST (IU/L)**	**ADP (ng/mg protein)**	**Body weight/g**
Control	11.98 ± 0.96	7.500 ± 0.057	43.75 ± 1.75	2.64 ± 0.100	19.70 ± 0.85	22.25 ± 0.85	25.65 ± 0.47	**196.30 ± 2.72**
HFCD	97.52 ± 2.38^a^	8.633 ± 0.033^a^	95.25 ± 2.52^a^	3.93 ± 0.08^a^	74.00 ± 3.6^a^	87.20 ± 4.1^a^	1.42 ± 0.039^a^	**214.00 ± 2.08^a^**
HFCD + cranberry 50 (50 mg/kg)	45.47 ± 0.97^a,b^	8.133 ± 0.033^ab^	59.25 ± 2.05^a,b^	3.29 ± 0.15^a,b^	37.50 ± 1.4^a,b^	48.00 ± 1.95^a,b^	13.51 ± 0.11^a,b^	**206.00 ± 0.57^a,b^**
HFCD + cranberry 100 (100 mg/kg)	22.89 ± 2.1^a,b^	7.66 ± 0.033^b^	40.25 ± 1.31^b^	3.07 ± 0.16^b^	26.50 ± 0.64^b^	31.50 ± 1.3^b^	20.57 ± 0.83^a,b^	**201.70 ± 0.88^b^**
Cranberry 100 (100 mg/kg)	9.79 ± 0.46^b^	7.433 ± 0.066^b^	33.25 ± 0.85^b^	2.29 ± 0.04^b^	17.20 ± 0.85^b^	18.50 ± 0.64^b^	30.14 ± 0.23^a,b^	**198.70 ± 0.88^b^**

**Rats were fed HFCD for 8 consecutive weeks with co-treatment of cranberry (50 and 100 mg/kg/day, orally, 3 times/week). Data are represented as mean ± S.E.M. (n = 10). ^*a,b*^Significantly different from control and HFCD group, respectively at P < 0.05, using ANOVA followed by Tukey–Kramer as a post hoc test. HCFD, high cholesterol fat diet; TG, triglyceride; ALT, alanine transaminase; AST, aspartate transaminase; HOMA-IR, homeostasis model assessment of insulin resistance; ADP, adiponectin.**

### Liver Histopathological Assessment

**FIGURE 2:**
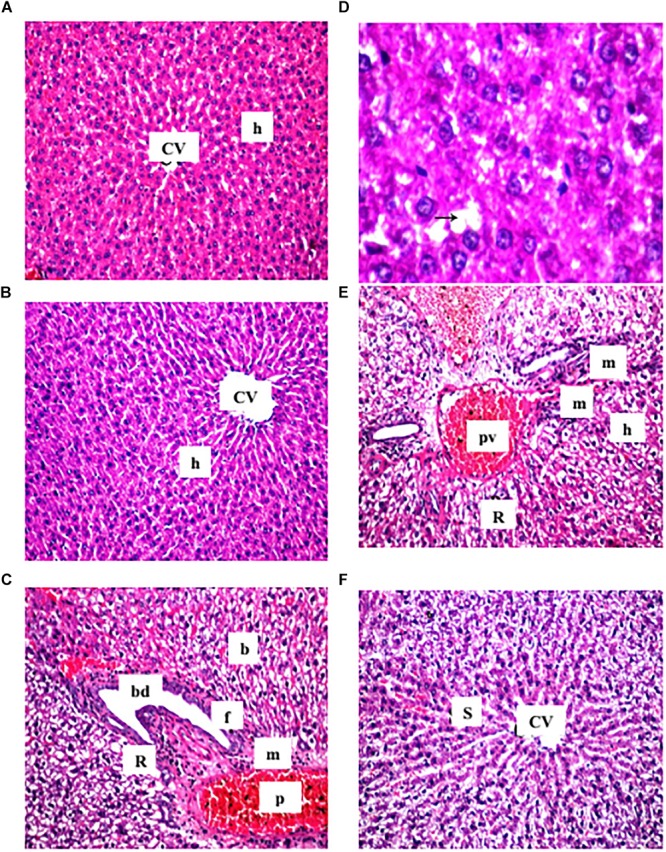
Representative photomicrographs of liver sections stained with hematoxylin and eosin (X40). **(A,B)** Sections taken from rat in the control and the cranberry-only groups, respectively showing normal histological structure of the central vein (CV) and surrounding hepatocyte (h). **(C)** Section taken from HFCD fed rats showing congestion in the portal vein (PV), fibrosis (F) with inflammatory cells infiltration (m) in the portal area associated with hyperplasia in the lining epithelium of bile duct (bd) as well as ballooning degeneration (b) and rupture (r) in the hepatocyte. **(D)** Section taken from HFCD rats showing lipid droplets accumulation (arrow) in the hepatocyte (X80). **(E)** Section taken from rats fed HFCD and co-treated with CE50 showing congestion in the portal vein (PV) and inflammatory cell infiltration (F) in the portal area as well as ballooning degeneration (b) in the hepatocyte. **(F)** Section taken from rats fed HFCD and co-treated with CE100 showing intact histological structure of the central vein and surrounding hepatocyte.

It was observed that the liver sections obtained from the control and cranberry-only treated groups showed normal hepatic architecture (Figures 2A,B). On the contrary, HFCD induced dilatation and congestion in both central and portal veins together with fibrosis and inflammatory cell infiltration, as well as hyperplasia in the lining epithelium of the bile ducts at the portal area. In addition, ballooning degeneration and rupturing of the hepatocyte were observed (Figures 2C,D). Meanwhile, the cranberry co-treatment (50 mg/kg) group still showed dilatation and congestion in the central and portal vein associated with ballooning degeneration in the hepatocyte, and the portal area had inflammatory cell infiltration (Figure 2E). Notably, the higher dose of cranberry (100 mg/kg) only showed mild congestion in the portal vein associated with mild ballooning degeneration in the hepatocytes and fortunately, with no signs of fibrosis (Figure 2F). Table 3 shows the NASH scoring in liver specimens taken from the different experimental groups.

**TABLE 3:** NASH scoring in liver specimens taken from the different experimental groups.

**Item**	**Definition**				
	**Control**	**HFCD**	**HFCD + Cranberry 50**	**HFCD + Cranberry 100**	**Cranberry 100**
**Steatosis**					
Grade	Nil	Severe	Mild	Nil	Nil
Location	–	Hepatic parenchyma	Hepatic parenchyma	–	–
Score	0	3	1	0	0
**Ballooning degeneration**					
Score	0	3	1	0	0
**Inflammation**					
Location	–	Portal area	Portal area	-	-
Score	0	2	1	0	0
Fibrosis score	0	3 portal	2 portal	0	0
Congestion	Absent	Present Central & portal veins	Present Central & portal veins	Present Central & portal veins	Absent
Hyperplasia	Absent	Present Bile duct	Absent	Absent	Absent
Microgranulomas	Absent	Absent	Absent	Absent	Absent
Acidophil bodies	Absent	Absent	Absent	Absent	Absent
Glycogenated nuclei	Absent	Absent	Absent	Absent	Absent
Mallory’s hyalline	Absent	Absent	Absent	Absent	Absent
Megamitochondria	Absent	present	Absent	Absent	Absent
Pigmented macrophage	Absent	Absent	Absent	Absent	Absent

### The Effect of Cranberry on Insulin Resistance

As shown in Table 2, HOMA-IR was significantly augmented in the HFCD group by 7-fold, as compared to the control group. On the other hand, this ration was notably reduced in rats given cranberry (50 and100 mg/kg) by 54 and 77%, respectively, as compared to the HFCD group. Moreover, TyG index was significantly augmented in the HFCD group by 15%, as compared to the control group. On the contrary, this index was notably reduced in rats given cranberry (50 and 100 mg/kg) by 6 and 11%, respectively, as compared to the HFCD group.

### The Effect of Cranberry on Oxidative Stress and Antioxidant Capacity

The effects of HCFD and/or cranberry on oxidative stress markers are shown in Table 4. There was a significant rise in the MDA level in the HFCD group by 10.4-fold, as compared to the control group. Co-treatment with cranberry (50 and100 mg/kg) significantly reduced this elevation by 44 and 64%, respectively, as compared to the disease group. Moreover, SOD and catalase activities as well as GSH tissue levels were significantly reduced in the HFCD group by nearly 80%, as compared to the control group. Nevertheless, the cranberry-administered rats showed a marked elevation in SOD, catalase activities as well as the depleted GSH. Notably, the higher dose of cranberry (100 mg/kg) significantly restored the depleted GSH by 257%, as compared to the HFCD group.

**TABLE 4:** The effect of cranberry nutraceutical (50 and 100 mg/kg) on oxidative stress markers, antioxidant enzymes, NF- κB levels in livers tissues, and serum levels of TNF-α and IL-6 in HFCD-induced liver fibrosis in rats.

**Groups**	**MDA (μmol/mg protein)**	**SOD (U/mg protein)**	**CAT (U/mg protein)**	**GSH (pg/mg protein)**	**IL-6 (pg/ml)**	**TNF-α (pg/ml)**	**NF- κB (ng/mg protein)**
Control	7.08 ± 0.10	137.00 ± 0.86	51.13 ± 0.61	43.22 ± 1.42	2.59 ± 0.008	32.70 ± 1.29	0.16 ± 0.003
HFCD	73.70 ± 2.00^a^	22.09 ± 0.58^a^	6.01 ± 0.05^a^	9.66 ± 0.60^a^	19.10 ± 0.13^a^	153.60 ± 6.5^a^	0.97 ± 0.014^a^
HFCD + cranberry 50 (50 mg/kg)	41.04 ± 0.37^a,b^	80.31 ± 2.72^a,b^	28.03 ± 1.45^a,b^	19.01 ± 1.33^a,b^	12.34 ± 0.56^a,b^	80.20 ± 5.2^a,b^	0.39 ± 0.02^a,b^
HFCD + cranberry 100 (100 mg/kg)	26.13 ± 1.63^a,b^	128.80 ± 0.54^a,b^	43.62 ± 1.19^a,b^	34.47 ± 0.74^a,b^	5.25 ± 0.25^a,b^	43.15 ± 1.40^b^	0.15 ± 0.003^b^
Cranberry 100 (100 mg/kg)	3.97 ± 0.23^b^	160.80 ± 1.19^a,b^	107.8 ± 3.25^b^	69.84 ± 3.34^a,b^	2.16 ± 0.002^b^	13.64 ± 1.44^a,b^	0.10 ± 0.001^b^

**Rats were fed HFCD for 8 consecutive weeks with co-treatment of cranberry* (*50 and 100 mg/kg/day, orally, 3 times/week).*Data are represented as mean ± S.E.M (n = 10). ^*a,b*^Significantly different from control and HFCD group, respectively at P < 0.05, using ANOVA followed by Tukey–Kramer as a post hoc test. HCFD, high cholesterol fat diet; MDA, malondialdehyde; SOD, superoxide dismutase; CAT, catalase; GSH, glutathione; TNF-α, tumor necrosis factor- alpha; IL-6, interleukin-6; NF- κb, nuclear factor-kappa B.***

### The Effect of Cranberry on Inflammatory Markers

As shown in Table 4, there was a significant rise in TNF-α and IL-6 level in the HFCD group by 3- and 6-fold, as compared to the control group. Cranberry (50 and 100 mg/kg) caused a significant lowering in the TNF-α level by 48 and 72%, respectively, as compared to the HFCD group. Additionally, IL-6 serum levels were significantly decreased by 36 and 73% in the groups co-treated with cranberry (50 and 100 mg/kg) respectively, as compared to the HFCD group. Furthermore, NF-κB is significantly raised in the HFCD group by 5-fold, as compared to the control group. As compared to the disease group, treatment with cranberry (50 and 100 mg/kg) markedly attenuated this elevation by 60 and 84%, respectively.

### The Effect of Cranberry on Insulin Signaling

**FIGURE 3:**
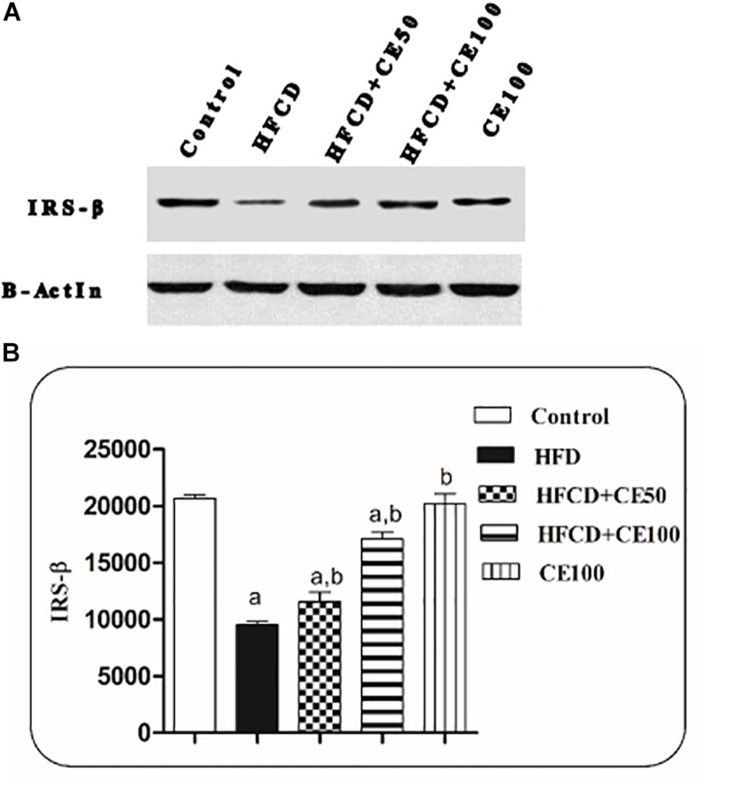
The effect of cranberry nutraceutical (50 and 100 mg/kg) on IRS-2 expression in HFCD-induced liver fibrosis in rats. **(A)** Western blot analysis of IRS-2 in rats fed HFCD and co-treated with cranberry 50 and/or cranberry 100. **(B)** Quantification of IRS-2 expression in rats fed HFCD and co-treated with cranberry 50 and/or cranberry 100. -Data are represented as mean ± S.E.M. (*n* = 5). (a) Significantly different from the control group and (b) significantly different from the HFCD groups, respectively, at *p* < 0.05 using ANOVA followed by Tukey–Kramer as a *post hoc* test.

Figure 3 shows that the expression of IRS-2 significantly reduced in the HFCD group by 54%, as compared to the control group. While in the cranberry-treated groups (50 and 100 mg/kg), IRS-2 expression was significantly increased by 21 and 79%, respectively, as compared to the HFCD group.

### The Effect of Cranberry on Cytoprotective Markers

**FIGURE 4:**
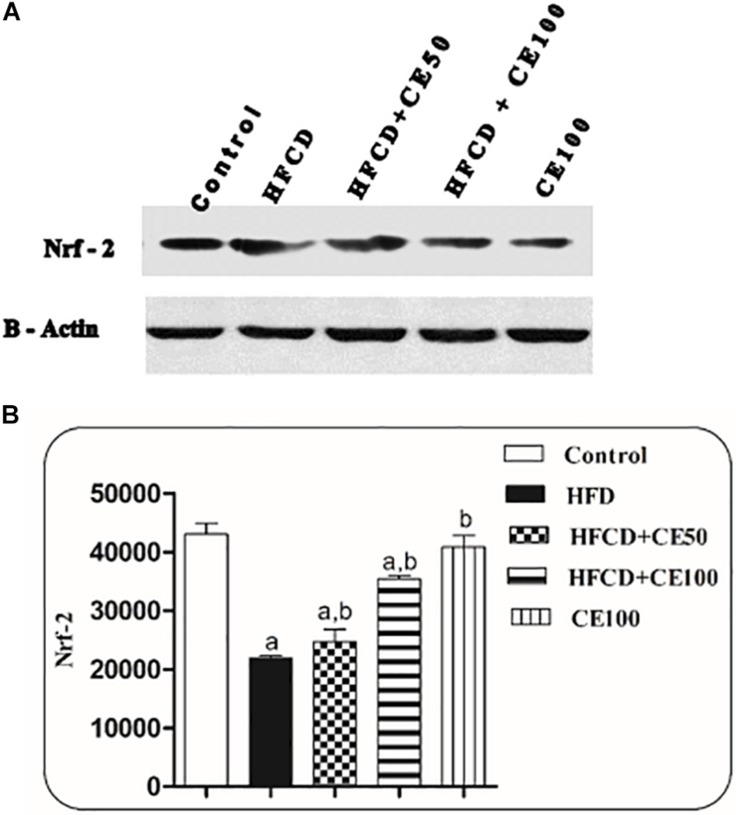
The effect of cranberry nutraceutical (50 and 100 mg/kg) on Nrf-2 expression in HFCD-induced liver fibrosis in rats. **(A)** Western blot analysis of nuclear Nrf-2 in rats fed HFCD and co-treated with cranberry 50 and/or cranberry 100. **(B)** Quantification of the nuclear Nrf-2 expression in rats fed HFCD and co-treated with cranberry 50 and/or cranberry 100. -Data are represented as mean ± S.E.M. (*n* = 5). a, b: Significantly different from the control and HFCD groups, respectively, at *p* < 0.05 using ANOVA followed by Tukey–Kramer as a *post hoc* test.

Indeed, HFCD induced a significant reduction in the ADP level by 95%, as compared to the control group. On the contrary, cranberry treatment (50 and 100 mg/kg) induced a marked elevation in ADP levels by 851 and 1348%, respectively, as compared to the HFCD group (Table 2). Furthermore, nuclear Nrf-2 was significantly decreased by 49% in the HFCD group, as compared to the control group. This decrease was significantly attenuated in cranberry-treated groups (50 and 100 mg/kg), where it was increased by 13 and 61%, respectively, as compared to the HFCD group (Figure 4).

### The Effect of Cranberry on Fibrotic Markers

**FIGURE 5:**
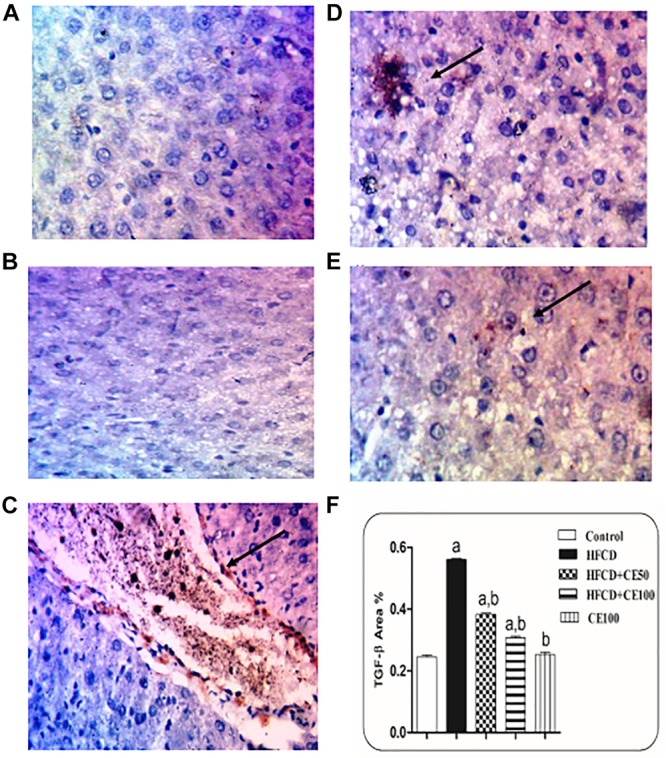
The effect of cranberry nutraceutical (50 and 100 mg/kg) on TGF-β expression in HFCD-induced liver fibrosis in rats by immunohistochemical staining. **(A,B)** Liver sections taken from rats in the control and the cranberry-only groups showing that TGF-β expression was minimal in the hepatic tissue. **(C)** Sections taken from rats in the HFCD group showing extensive TGF-β expression (brown color). **(D)** Sections taken from rats fed HFCD and co-treated with cranberry 50 showing mild TGF-β expression. **(E)** Sections taken from rats fed HFCD and co-treated with cranberry 100 showing minimal TGF-β expression (brown color). All sections were magnified at (X200). **(F)** Quantitative image analysis expressed as percentage of area of immunopositive reaction. Data are represented as mean ± S.E.M. (*n* = 5). a, b: Significantly different from the control and HFCD groups, respectively, at *p* < 0.05 using ANOVA followed by Tukey–Kramer as a *post hoc* test.

**FIGURE 6:**
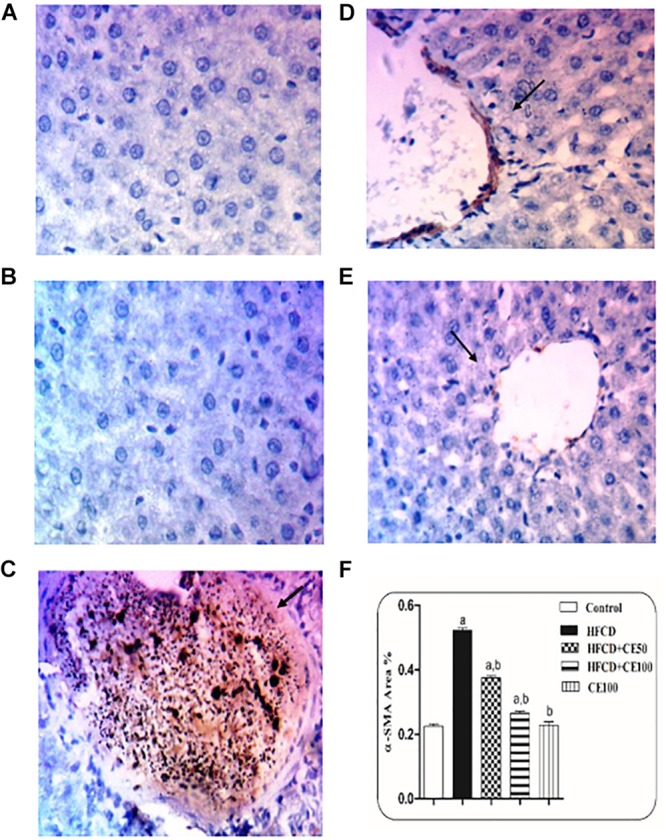
The effect of cranberry nutraceutical (50 and 100 mg/kg) on α-SMA expression in HFCD-induced liver fibrosis in rats by immunohistochemical staining. **(A,B)** Liver sections taken from rats in the control and the cranberry-only groups showing minimal expression of α-SMA. **(C)** show sections taken from rats in the HFCD displaying extensive α-SMA expression (brown color). **(D)** Sections taken from the group fed HFCD and co-treated with cranberry 50 showing mild α-SMA expression. **(E)** Sections taken from rats fed HFCD and co-treated with cranberry 100 showing minimal α-SMA expression (brown color). All sections were magnified at (X200). **(F)** Quantitative image analysis expressed as percentage of area of immunopositive reaction. Data are represented as mean ± S.E.M. (*n* = 5). a, b: Significantly different from the control and HFCD groups, respectively, at *p* < 0.05 using ANOVA followed by Tukey–Kramer as a *post hoc* test.

**FIGURE 7:**
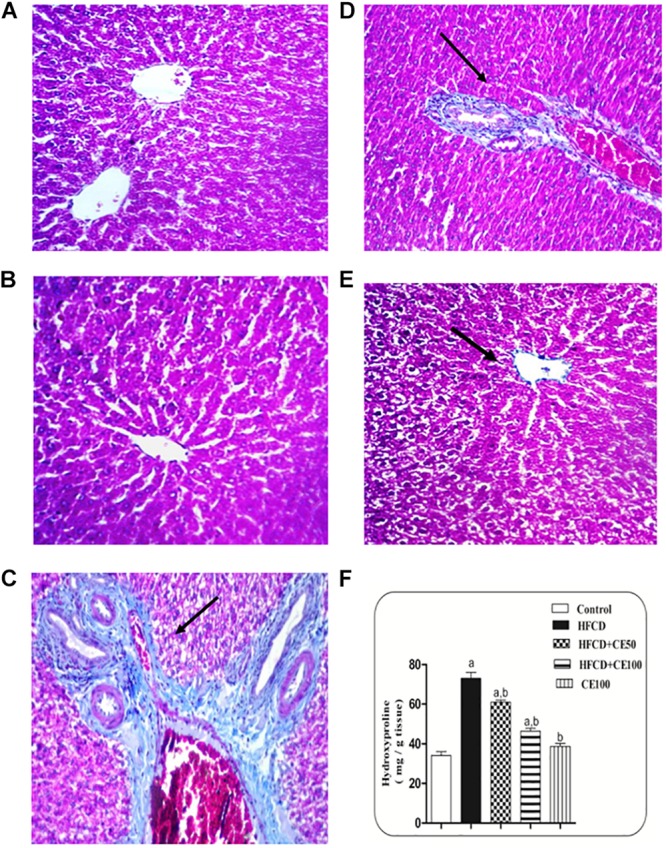
Representative photomicrographs of liver sections stained with Masson’s trichrome. **(A,B)** hepatic sections taken from rat in the control and the cranberry-only groups, respectively showing normal histological structure in the portal area with minimal collagen deposition. **(C)** Section taken from HFCD rats showing severe congestion in the portal vein and excessive collagen fibers deposition along with pseudolobules formation in the portal area. **(D)** Section taken from rats fed HFCD and co-treated with cranberry 50 showing nearly an absence of pseudolobules with less collagen deposition. **(E)** Section taken from rats fed HFCD and co-treated with cranberry 100 showing an absence of pseudolobules and minimal collagen fibers deposition. All sections were magnified at (X40). **(F)** Liver hydroxyproline content in different groups, Data are represented as mean ± S.E.M. (*n* = 5). a, b: Significantly different from the control and HFCD groups, respectively, at *p* < 0.05 using ANOVA followed by Tukey–Kramer as a *post hoc* test.

As compared to the control group, TGF-β was significantly increased the HFCD group by 1-fold. In contrast, cranberry significantly decreased this ratio by 28 and 49%, at doses of 50 and 100 mg/kg, respectively, as compared to HFCD group (Figure 5). In addition, α-SMA increased significantly in the HFCD group by 1-fold, as compared to the control group. Co-treatment with cranberry (50 and 100 mg/kg) significantly decreased α-SMA tissue levels by 32 and 45%, respectively, as compared to the HFCD group (Figure 6). Additionally, there was a significant increase in the hydroxyproline level in the HFCD group by 114%, as compared to the control group. Cranberry, at doses of 50 and100 mg/kg, caused a significant reduction in hydroxyproline levels by 17 and 37%, respectively, as compared to the HFCD group. Furthermore, Figure 7 represents the Masson trichrome staining of liver tissue. Sections taken from the control and cranberry 100 only treated group showed minimal blue staining in comparison to the HFCD group. In contrast, the HFCD group showed an extensive blue color that presented between the hepatic lobules, indicating a strong positive histochemical reaction for collagen fibers (Figure 7C), Meanwhile, rats fed with HFCD and co-treated with 50 mg/kg cranberry showed mild staining, indicating a reduction of collagen deposition (Figure 7D). Moreover, rats fed with HFCD and co-treated with 100 mg/kg cranberry showed limited staining, indicating more reduction of collagen deposition (Figure 7E).

## Discussion

Non-alcoholic fatty liver disease is a metabolic disorder associated with the accumulation of fat in the hepatocyte together with inflammation and IR (Than and Newsome, 2015). The reduction in the levels of the cytoprotective ADP (Hotta et al., 2000; Tsochatzis et al., 2009) and Nrf-2 (Chowdhry et al., 2010; Shin et al., 2013) also contributes to the progression to the liver fibrosis stage (Contos et al., 2004). There is increasing evidence that the ingestion of fruits have many benefits in reducing the risk of MS (Morimoto et al., 2012; Chan et al., 2013). Consequently, this study was conducted to investigate, for the first time, the possible anti-fibrotic effect of cranberry nutraceutical in a model of high fat diet induced NAFLD and liver fibrosis in rats. Moreover, the possible ameliorative effect of cranberry was clarified *via* studying different oxidative, inflammatory markers as well as insulin sensitivity and signaling.

Cranberries are considered a rich source of phenolic acids, including hydroxybenzoic and hydroxycinnamic acid derivatives, which impart the berries with a unique flavor (Vattem et al., 2005). Moreover, cranberries are rich in flavonoids and proanthocynidins (Borges et al., 2010). In the current study, cranberry nutraceutical was characterized using UPLC-ESI-MS/MS. In addition, the total flavonoids and the total phenolic content in cranberry extract were quantified as quercetin and gallic acid equivalents, respectively.

First, to confirm the hepatoprotective effects of cranberry, liver function tests were performed. The results of our study showed that ALT and AST significantly increased in the HFCD group as compared to the control group, as previously shown (Chen et al., 2015). Co-treatment with cranberry significantly reduced AST and ALT serum levels. The hepatoprotective effect of cranberry was previously shown in different models; myocardial necrosis (Ali et al., 2015) as well as the liver mitochondrial damage model (Cheshchevik et al., 2012) with a more obvious effect at higher doses of cranberry (100 mg/kg).

These results were confirmed by histopathological examination of liver sections taken from different treatment groups. Our model showed marked dilatation and congestion in both central and portal veins associated with fibrosis and inflammatory cell infiltration in HFCD-fed rats. This was in addition to the ballooning degeneration and rupture of the hepatocytes. Both doses of cranberry markedly ameliorated these changes in the current study. Notably, cranberry has the ability to ameliorate most of the features of NAFLD, from histological changes to fibrosis, where the higher dose (100 mg/kg) provided more hepatoprotection.

Hepatic TGs buildup is a measure of steatosis, which if combined with inflammation and oxidative stress may lead to NAFLD (Anhê et al., 2014). Moreover, hepatic steatosis is considered a benign case, but growing evidence proposes that TGs accretion sensitizes the hepatocyte fibrosis and inflammation (Day and James, 1998). As compared to the HFCD group, co-treatment with cranberry, at the dose of 100 mg/kg, showed a more significant decrease in the level of TGs than cranberry 50. The TGs-lowering ability of cranberry were proven with comparison to HFCD group, where co-treatment with cranberry at dose 100 mg/kg showed more significant decrease in TGs level than the dose of 50 mg/kg. These results were shown in diet induced-obesity in a mice model (Anhê et al., 2014) and metabolic syndrome model (Khanal et al., 2010).

Indeed, insulin resistance has been strongly associated with hepatic fat accumulation (Utzschneider and Kahn, 2006; Petta et al., 2009), as peripheral IR increases lipolysis and increases the transfer of FFA to the liver tissue which is stored as TG (Berlanga et al., 2014). Moreover, it increases DNL in the hepatocytes (Bajaj et al., 2004). In the present study, HFCD caused a significant increase in the HOMA-IR, which is in alignment with a previous study carried out by Chen et al., 2015. Different doses of cranberry markedly improved insulin sensitivity based on decreasing HOMA-IR, where cranberry 100 provided a more significant improvement of insulin sensitivity. These results are in alignment with those obtained by Anhê et al. (2014) and Khanal et al. (2010).

The Triglyceride glucose index (TyG index) seems to be an interesting marker to predict NASH or fibrosis in obese patients, Moreover, it has been recognized as an insulin resistance marker (Zhang et al., 2017). In the HFCD group, the level of the TyG index was significantly raised in comparison to the control group. As compared to the HFCD group, co-treatment with cranberry, at the dose of 100 mg/kg, showed a more significant decrease in the level of the TyG index than cranberry 50.

As previously discussed, IR, which is considered a key feature of MetS, plays a crucial role in the progress of NAFLD. Several studies revealed that hepatic IR correlates with increased liver fat content (Camporez et al., 2013; Cantley et al., 2013). In this context, inhibition of IRS-2 is considered a major cause of hepatic IR which results in complete loss of insulin signaling events, ending with inhibition of insulin action on the content. Supporting the hypothesis that any defect in insulin signaling lead to IR, in this context, inhibition of IRS-β is considered as a major cause of hepatic IR which results in complete loss in insulin signaling events, ending with inhibition of insulin action on the liver. This, in turn, results in failure to suppress gluconeogenesis, promote glycogen storage, and to increase hepatic glucose uptake (Michael et al., 2000; Shimomura et al., 2000). Moreover, it has been reported that IR and prolonged hyperinsulinemia are associated with an increase in the expression of the transcription factor sterol regulatory element binding protein 1C (SREBP-1c), resulting in increasing DNL. Moreover, SREBP-1c defeats IRS-2-mediated insulin signaling *via* direct interaction with its promoter (Ide et al., 2004). In addition, the up-regulation of SREBP-1c as a result of knockdown of IRS-2 leads to an increase in DNL, accumulation of fatty acids in hepatocytes, and finally as a net result exacerbation of steatosis (Kohjima et al., 2008). In the present study, HFCD caused a significant decrease in IRS-2 expression, these findings were confirmed by Xing et al. (2011). Both doses of cranberry caused a significant increase in IRS-2 expression, with more obvious effect at the higher dose of cranberry.

Since the liver is considered as a metabolically active organ, it is responsible for the biotransformation and detoxification of xenobiotics. It is therefore particularly susceptible to oxidative stress (Levonen et al., 2014), which, in turn, is implicated in the pathogenesis of NAFLD (Chambel et al., 2015). Our current study confirmed that HFCD induces significant oxidative stress with a marked decrease in the total antioxidant profile. This is in alignment with a number of studies which show that there is an increase in the level of free radicals in the rat model of NASH, while the level of anti-oxidant defense mechanisms, such as GSH, are decreased (Grattagliano et al., 2000).

Any defect in lipid metabolism may cause lipid peroxidation which is the main cause leading to oxidative stress and inflammation, whereas ROS increase cytokine secretion from hepatocyte leads to the beginning of immune-arbitrated mechanisms contributing to further liver damage (Than and Newsome, 2015). TNF-α is an important pro-inflammatory adipocytokines, and was identified as the first inflammatory molecule linked with IR (Lang et al., 1992). Interestingly, a pharmacological decrease in TNF-α has been associated with the improvement of NAFLD in some patients (Satapathy et al., 2004). High serum level of TNF-α have been found in patients with NASH (Tsochatzis et al., 2009; Hebbard and George, 2011; Berlanga et al., 2014). This is confirmed by our study results. These changes are markedly ameliorated at both doses of cranberry. These results are in alignment with the previous study carried out by Huang et al. (2009) in *Escherichia coli*, with more obvious effects at a dose of 100 mg/kg.

Indeed, the pleiotropic cytokine; IL-6, is expressed in many inflammatory cells, regulating a number of biological processes including both inflammation and IR (Berlanga et al., 2014). Interleukin-6 depletion was predicted to improve insulin responsiveness in both adipose tissue and liver (Klover et al., 2005). The role of IL-6 in antagonizing the insulin receptor signal transduction in the hepatocyte is a result of the decrease of IRS expression (Rotter et al., 2003). In the present work, HFCD caused a significant increase in serum IL-6. These findings were confirmed by previous work (Haukeland et al., 2006; Tsochatzis et al., 2009). Both doses of cranberry caused a significant decrease in the serum level of IL-6, where cranberry 100 caused a more significant decrease in the IL-6 level. The ability of CR to suppress the expression of IL-6 was shown in other models (Bodet et al., 2007; Huang et al., 2009).

A central regulator of the metabolic inflammatory signaling pathway in the liver is NF-κB, which controls the production of many pro-inflammatory cytokines including TNF-α and IL-6 (Anhê et al., 2014). Moreover, NF-κB modulates hepatic fibrogenesis by regulating hepatocyte injury, which is considered the primary cause of fibrogenic responses in the hepatocyte (Luedde and Schwabe, 2011). In the present study, HFCD caused a significant increase in hepatic NF-κB expression as shown in previous work (Frantz, 2005). Co-treatment with both doses of cranberry resulted in a significant decrease in hepatic NF-κB expression with more effects at the higher dose of 100 mg/kg. These results were supported in P-fimbriated *Escherichia coli* (Huang et al., 2009) and in diet induced-obesity in gut microbiota in a mouse model (Anhê et al., 2014).

In addition, fatty liver is usually associated with low levels of the cytoprotective marker ADP, which is one of the main products of adipocyte. Adiponectin was shown to have anti-inflammatory and anti-diabetic effects (Berlanga et al., 2014). Several studies have suggested that ADP stimulates the release of anti- inflammatory cytokines, blocks NF-κB activation, and inhibits the release of TNF-α and IL-6 (Wolf et al., 2004; Tilg and Moschen, 2006). Moreover, it alleviates hepatic steatosis inflammation *via* the inhibition of the hepatic TNF-α production. It also ameliorates the increase of transaminases levels by accelerating fatty acid oxidation in the hepatocyte and inhibits the activities of fatty acid synthesis enzymes (Xu et al., 2003). It also prevents fibrosis, which is partially mediated by reducing the production of TGF-β (Kamada et al., 2003). Previous studies state that the serum level of ADP was lower in a NASH model compared to the control group (Hotta et al., 2000; Tsochatzis et al., 2009). These results were confirmed by our study, as HFCD caused a significant decrease in the hepatic ADP levels compared to the control group. Both doses of cranberry significantly increased the level of the hepatic ADP, in a dose-dependent manner. These results are in alignment with previous studies (Kowalska et al., 2015), where cranberries inhibited lipid metabolism and modulated adiponectin secretion in a 3T3-L1 adipocytes model, and cranberry juice increased adiponectin levels in patients with metabolic syndrome (Lozovoy et al., 2013).

It is recognized that the transcription factor Nrf-2, contributes to cellular defense mechanisms by down-regulating the genes that promote lipid accumulation in the liver so it may protect the hepatocyte from further damage perpetrated by TGs accumulation. It also activates the antioxidant genes and electrophiles derived from lipid peroxidation, consequently preventing mitochondrial dysfunction and liver oxidative stress (Chambel et al., 2015). Furthermore, a previous study revealed that Nrf-2 activation attenuate TGF-β induced SMAD-3 phosphorylation and collagen expression which in turn attenuates the fibrosis (Oh et al., 2012). In agreement with previous studies carried out by Chowdhry et al. (2010) and Shin et al. (2013), HFCD showed a significant reduction in the hepatocyte level of nuclearNrf-2. Both doses of cranberry caused a significant increase in the hepatocyte level of nuclearNrf-2, where the higher dose showed better results.

Fibrosis is considered to be one of the main features of NAFLD. Liver fibrosis is now considered to be a reversible process, where excessive extracellular matrix (ECM) is accumulated. The therapeutic options for liver fibrosis are limited. It is worth mentioning that untreated liver fibrosis will definitely progress into cirrhosis and liver cancer, ending in liver failure. Indeed, HSCs are the central mediators of hepatic fibrosis. Lipid peroxidation can promote HSCs proliferation, which is activated during liver injury to collagen-producing HSCs. These cells, subsequently release a great number of cytokines, among which TGF-β is recognized as the chief fibrogenic cytokine. Oxidative stress has been shown to directly activate TGF-β (Barcellos-Hoff and Dix, 1996; Jobling et al., 2006). Previous studies revealed that TGF-β induces α-SMA expression, contributing to further liver fibrosis (Desmoulière et al., 1993; Goldberg et al., 2007). In the present work, HFCD caused a significant rise in the hepatic levels of TGF-β and α-SMA, together with excessive collagen fiber deposition show that liver fibrosis was evident. Meanwhile, the co-treatment with cranberry markedly attenuated this effect, in a dose-dependent manner. Moreover, the anti-fibrotic effect of cranberry was further confirmed by masson’s trichrome staining as well as the biochemical assessment of collagen.

## Conclusion

In conclusion, cranberry provided hepatoprotection against the model that induced NAFLD in rats. Cranberry significantly reduced hepatic steatosis as well as oxidative stress. Cranberry also markedly ameliorated the inflammatory status, which was demonstrated by the decreasing expression of TNF-α, IL-6, and NF-κB. The anti-inflammatory and anti-fibrogenic cytokine; ADP, was also significantly induced by cranberry administration. Notably, insulin sensitivity was improved on cranberry consumption, as evidenced by improving HOMA-IR and up-regulated IRS-2 expression. Beside all these findings, the ability of cranberry to enhance Nrf-2 expression in the hepatic tissues was shown to be partially involved in its hepatoprotective properties. Most importantly, this was the first study to show that cranberry can markedly ameliorate liver fibrosis development in a NAFLD model, where collagen production and the expression of the fibrotic markers (TGF-β and α-SMA) were significantly reduced. Accordingly, the consumption of cranberry may be beneficial in delaying the initiation and progression of NAFLD as well as liver fibrosis.

## Data Availability Statement

All datasets generated for this study are included in the article/supplementary material.

## Ethics Statement

The animal study was reviewed and approved by the Research Ethics Committee of Ain Shams University (REC-ASU), Egypt.

## Author Contributions

SF, NS, RE-N, and SA designed the experiments and wrote the manuscript. SF performed the experiments and provided the reagents and the materials. SF, NS, and RE-N analyzed the data. IA performed the herbal extract standardization and LCMS characterization.

## Conflict of Interest

The authors declare that the research was conducted in the absence of any commercial or financial relationships that could be construed as a potential conflict of interest.

## Abbreviations

ADP: Adiponectin
CE: Cranberry powder extract
DNL: *De novo* lipogenesis
FFAs: Free fatty acids
GSH: Glutathione
HFCD: High fat cholesterol diet
HOMA-IR: Homeostasis model assessment insulin resistance index
HRP: horseradish peroxidase
HSCs: Hepatic stellate cells
IL-6: Interleukin-6
IR: Insulin resistance
IRS-β: Insulin receptor substrate beta
MetS: Metabolic syndrome
NAFLD: Non-alcoholic fatty liver disease
NASH: Non-alcoholic steatohepatitis
NF-_k_B: Nuclear factor kappa b
Nrf-2: Nuclear factor erythroid-2-related factor-2
ROS: Reactive oxygen species
SDS: sodium dodecyl sulfate
SOD: Super oxide dismutase
SREBP-1C: Sterol regulatory element binding protein 1C
TGF-β: growth factor beta
TNF-α: Tumor necrosis factor –alpha
α-SMA: smooth muscle actin
